# Environmental predictors impact microbial-based postmortem interval (PMI) estimation models within human decomposition soils

**DOI:** 10.1371/journal.pone.0311906

**Published:** 2024-10-11

**Authors:** Allison R. Mason, Hayden S. McKee-Zech, Dawnie W. Steadman, Jennifer M. DeBruyn

**Affiliations:** 1 Department of Microbiology, University of Tennessee-Knoxville, Knoxville, TN, United States of America; 2 Department of Anthropology, University of Tennessee-Knoxville, Knoxville, TN, United States of America; 3 Department of Biosystems Engineering and Soil Science, University of Tennessee-Knoxville, Knoxville, TN, United States of America; Free University of Bolzano: Libera Universita di Bolzano, ITALY

## Abstract

Microbial succession has been suggested to supplement established postmortem interval (PMI) estimation methods for human remains. Due to limitations of entomological and morphological PMI methods, microbes are an intriguing target for forensic applications as they are present at all stages of decomposition. Previous machine learning models from soil necrobiome data have produced PMI error rates from two and a half to six days; however, these models are built solely on amplicon sequencing of biomarkers (e.g., 16S, 18S rRNA genes) and do not consider environmental factors that influence the presence and abundance of microbial decomposers. This study builds upon current research by evaluating the inclusion of environmental data on microbial-based PMI estimates from decomposition soil samples. Random forest regression models were built to predict PMI using relative taxon abundances obtained from different biological markers (bacterial 16S, fungal ITS, 16S-ITS combined) and taxonomic levels (phylum, class, order, OTU), both with and without environmental predictors (ambient temperature, soil pH, soil conductivity, and enzyme activities) from 19 deceased human individuals that decomposed on the soil surface (Tennessee, USA). Model performance was evaluated by calculating the mean absolute error (MAE). MAE ranged from 804 to 997 accumulated degree hours (ADH) across all models. 16S models outperformed ITS models (p = 0.006), while combining 16S and ITS did not improve upon 16S models alone (p = 0.47). Inclusion of environmental data in PMI prediction models had varied effects on MAE depending on the biological marker and taxonomic level conserved. Specifically, inclusion of the measured environmental features reduced MAE for all ITS models, but improved 16S models at higher taxonomic levels (phylum and class). Overall, we demonstrated some level of predictability in soil microbial succession during human decomposition, however error rates were high when considering a moderate population of donors.

## Introduction

Microbial communities undergo succession in response to disturbance events [1]. Vertebrate death and subsequent decomposition represent one such event, where microbial community composition is altered in response to nutrient deposition and altered environmental conditions [2–7]. Microbial succession has been studied in various carcass/cadaver decomposition microhabitats, including internal organs [8–14], skin [3, 4, 15–18], bone [19, 20], and soils [4, 5, 18, 21, 22]. These studies suggest that these successional changes may be robust and universal enough to be used to predict the postmortem interval (PMI), or time elapsed since death (or beginning of decomposition). PMI estimations can be important evidence for death investigations, allowing law enforcement to establish a timeline of events [23]. The work developing microbial-based PMI models has been inspired by forensic entomology methods, which link insect succession or development to PMI. Entomological PMI estimation methods are widely used, but limited to forensic cases where insects are present, the species is identifiable, and temperature data can be collected. Unlike insects, microbes do not have a pre-colonization phase where they must be exposed to, detect, and accept a carcass [24, 25], as they are host-associated and present in the surrounding environment [7]. Together, this makes microbes advantageous for developing a forensic application estimating time since death.

Thus far researchers have assessed microbial abundance-based PMI prediction models in all major microhabitats and three different mammalian species. This includes internal [26, 27], external/skin [3, 17, 18, 28, 29], and soil [28, 29] microbial communities during pig (*Sus scrofa*) [3], mouse (*Mus musculus*) [18, 26, 28], and human (*Homo sapiens*) [17, 18, 27–29] decomposition. These models suggest some level of predictability in microbial succession; however, PMI estimations differ based on the microhabitat, taxonomic level considered, and algorithm used for model construction. To date, most studies apply supervised machine learning algorithms to taxonomic abundance data derived from amplicon sequencing of conserved markers of a few taxa [3] or whole microbial communities [17, 18, 26–29]. Within these studies, random forest regression is the most frequently used supervised machine learning algorithm. These microbial PMI models report error ranging from ~15 hours for mouse intestine samples to ~58 hours in mouse brain samples [26], up to 138 accumulated degree days for human skin samples [17], and two to six days for soil samples below decomposing mice and humans [28].

Of the three decomposition microhabitats in terrestrial decomposition systems (*i.e*., internal, external, and soil), soils have received the least attention. While there have been multiple studies assessing internal and external/skin succession [3, 17, 18, 26–28], only two studies have included swabs of the soil surface (*i.e*., O horizon) during mouse and human decomposition, demonstrating repeatable succession [28, 29]. It is unknown if these PMI models could be applied to microbiological changes within mineral soil horizons. Further, it is unclear how inter-individual variation and potential species differences may impact model performance, and thus what the predictability would be across a large population of humans. Recent work suggests differences in decomposition patterns may exist between species [30, 31] and even within species, due to intrinsic carcass properties (*e.g*., body composition) [22]. Consequently, there is a need to investigate the predictability of soil microbial succession within larger human sample sizes in order to assess applicability of these models for forensic science. Additionally, current microbial-based PMI estimation models are trained using relative abundance of microbial taxa as model features. However, observed changes in soil environmental parameters over time may also be used as indicators of decomposition time. For example, soil electrical conductivity (correlates to salinity), ammonium, and nitrate concentrations have been shown to undergo predictable changes in human decomposition soils [32–34]. Thus, it is possible that the inclusion of environmental predictors along with taxon relative abundance may help to improve model estimation.

The goals of this study were to 1) determine the utility of soil microbial communities for predicting decomposition time during human decomposition using 19 replicate human donors at a single location (East Tennessee); 2) determine which biological marker (16S rRNA, Internal Transcribed Spacer (ITS), or both) and taxonomic level (phylum, class, order, or OTU) results in the most accurate model predictions; and 3) assess how inclusion of soil environmental parameters (e.g., moisture, temperature, pH, conductivity, and enzyme rates) as model features affects model accuracy. Our first aim was to investigate microbial-based model performance across a human sample set (n = 19) collected in East Tenneessee to validate previously developed models. Our second aim was to evaluate model performance using different biological markers, *i.e*., genes commonly used as sequencing targets. Previous work reported that 16S rRNA gene-based models performed better then 18S rRNA gene or ITS models from organic residues collected on soil surfaces [28, 29]. However, we hypothesized that ITS-based models would be more accurate than 16S-based models due to our previous obervations showing that fungal community composition between individuals became more similar over decomposition time, whereas bacterial communities did not, suggesting less noise and better predictability in the fungal communities [22]. We also aimed to assess which taxonomic level(s) resulted in the greatest model accuracy. Based on previous results [22, 28]), we hypothesized that higher taxonomic levels, such as phylum and class would provide better PMI prediction. Our third aim was to probe the impact of environmental features on model prediction. While no previous studies have addressed this question, we hypothesized that inclusion of environmental predictors known to change in decomposition soils would help to improve PMI model predictions. Soil pH and conductivity are known drivers of microbial community dynamics [35, 36], while enzyme activities provide insight into functionality of the microbial community, so we chose to evaluate these parameters. We addressed our study aims using sequencing (16S rRNA and ITS2 amplicon) and soil physicochemical data from 19 deceased human individuals [22] decomposed on the soil surface at the University of Tennessee’s Anthropology Research Facility (ARF) in Tennessee, USA. Random forest regressions were applied to datasets with different combinations of biological markers (16S only, ITS only, 16S and ITS combined) and taxonomic levels (phylum, class, order, OTU), both with and without environmental predictors. Model performance was then compared by calculating the mean absolute error (MAE) to determine the influence of different combinations of features on PMI estimation.

## Materials and methods

### Study design

This work uses datasets generated from our previous study [22], which revealed the influence of intrinsic, or cadaver-related factors, on explaining variation in soil microbial communities during human decomposition. The current study, however, uses these datasets to assess the effects of environmental factors on predictability of this succession to estimate PMI. Full experimental details are reported in [22]. Briefly, decomposition of 19 deceased whole body human donors took place at the Anthropology Research Facility (ARF), located at the University of Tennessee in Knoxville, TN, USA (35°56’ 28” N, 83°56’ 25” W). The ARF is a forested outdoor facility consisting of clay loam and channery clay loam soils of the Coghill-Corryton complex (CcE) [19, 37].

Adult individuals with no open wounds or had not been autopsied were chosen for this study, as this could alter microbial decomposers prior to and during our study. Individuals were selected independent of demographic categories, however all individuals self-identified as White and ranged in age from 40 to 91 years (S1 Table) [22]. All individuals were whole body donors to the Forensic Anthropology Center (https://fac.utk.edu/body-donation/) specifically for the purpose of decomposition research. No living human subjects were involved and only donors who consent to decomposition research on their donation paperwork were enrolled in this study. The University of Tennessee, Knoxville, Human Research Protections Program (HRPP) reviewed this project and determined that research with human donors is exempt under 45 CFR 46.101. Individuals were placed supine unclothed on the soil surface between February 2019 and March 2020 (S1 Table). Hourly temperatures were recorded using TinyTag temperature and humidity loggers (Gemini Data Loggers, UK) until un-enrollment at the end of active decomposition, characterized by collapse of the abdomen and cessation of fluid leaking from the trunk [38]. Accumulated degree hours (ADH) were calculated using hourly temperature readings: 0 ADH was defined as time of placement within ARF, and a baseline temperature of 10°C was used for ADH calculations to keep our results comparable with entomology-based methods [39].

### Soil sampling and analysis

Five-cm soil cores were collected from the decomposition-impacted area surrounding each individual (within ~ 7.6 cm of the body), as well as from control sites located at least 1 m away from the donor (either upslope or at the same elevation) at predetermined accumulated degree hour (ADH) intervals until the end of active decomposition [22]. ADH intervals included 0 (prior to placement), 100, 250, 500, 750, and 1000 ADH, and thereafter at 500 ADH intervals until un-enrollment. For each respective sample, cores were homogenized and debris (*e.g*., roots, insect larvae, rocks, etc.) removed by hand. A subset of soils (~ 20 g) were stored in a 4 oz. Whirl-Pak bag (Nasco), flash frozen in liquid nitrogen and stored at -80°C prior to DNA extraction and extracellular enzyme assays. The remaining soil was was stored in a 7 oz. Whirl-Pak bag (Nasco) at 4°C for soil physiochemical measurements [22]. Soil slurries were prepared as a 1:2 ratio of soil to deionized water, allowed to come to room temperature for 30 minutes, and soil pH and electrical conductivity (EC) were measured using an Orion Star^™^A329 pH/ISE/Conductivity/Dissolved Oxygen portable multiparameter meter (ThermoFisher). Gravimetric soil moisture was measured in duplicate by oven drying 2 to 3 g soil aliquots at 105°C for 72 hours. Enzyme activities of *β*-glucosidase (BG), N-acetyl-*β*-D-glucosaminidase (NAG), leucine amino peptidase (LAP), and alkaline phosphatase (PHOS) were measured according to a modified procedure by Bell et al. (2013) [22, 40]. Breifly, 2.75 g of soil was weighed from soils stored at -80°C and held at -20°C until assays. Soils were thawed at room temperature prior to slurrying in 50 mM Tris buffer at pH 6.7 in a blender (Waring commercial blender, model WF2212114). Assays were conducted in triplicate using 800 μl of slurry and 200 μl of enzyme substrate (1,500 μM). Standard curves (MUB or MUC) were evaluted for each plate with conentrations ranging from 0 μM to 200 μM [22].

### DNA extraction, sequencing, and amplicon sequence analysis

DNA was extracted from soils stored at -80°C [22]. Briefly, 0.25 g of soil was extracted with the DNeasy Powerlyzer PowerSoil kit (QIAGEN Inc.) following manufacturer’s instructions with modifications for our soil texture (clay loam) and condition (high organic content). Specifically, soils were homogenized under parameters suggested for high organic soils (2,500 RPM for 45 s). DNA concentration was determined using a fluorometric assay (Quant-iT^™^ PicoGreen^®^ dsDNA Assay Kit, Invitrogen) with a total volume of 200 *μ*l and 1 *μ*l of DNA. All DNA extracts were sent to the University of Tennessee Sequencing Core Facility (Knoxville, TN) for 16S rRNA and ITS2 region amplicon sequencing on the Illumina MiSeq platform (2 x 150 bp). The primer set 515F [41] /806R [42] was used to amplify the V4 region of the 16S rRNA gene, while the ITS2 region in fungi was amplified using a mixture of primers (6 forward and 2 reverse: ITS3NGS1, ITS3NGS2, ITS3NGS3, ITS3NGS4, ITS3NGS5, ITS3NGS10, ITS4NGR, and ARCH-ITS4) described previously [43]. All raw sequences have been deposited in the National Center for Biotechnology Information’s Sequence Read Archive under the BioProject PRJNA817528.

Raw sequences were processed in Mothur [44] (v.1.43.0) to cluster into 97% similarity operational taxonomic units (OTUs) and generate OTU count tables for both 16S and ITS datasets as described in [22]. Briefly, paired-end reads were combined into contings, removing low-quality sequences (16S: Q > 20, bp ≤ 50; ITS Q > 20, bp < 200), sequences with ambigious bases (≥ 1), and primers/adpaters. Chimeras were removed using VSEARCH. Remaining sequences were classified using the SILVA non-redundant database [45] (v132) or UNITE RefS database [46] (version 02.02.2020) for 16S and ITS sequences, respectively. Bacterial sequences were then clustered into OTUs based on ≥ 97% sequence similarity and the Mothur default method, opticlust, while fungal sequences were clustered using abundance-based greedy clustering. We chose to cluster our sequences into OTUs rather than ASVs to reduce dimensionality in our dataset and the probability of splitting single genomes across multiple ASVs [47], especially when considering the diversity expected across soil microbial genomes. Count tables were then exported for analysis in R (version 4.4.0). Control samples (*e.g*., those not exposed to decomposition) and samples greater than 5000 ADH were removed using phyloseq [48] (v1.44.0). Samples were cut off at 5000 ADH to capture the linear response of soil parameters and account for variation in decomposition timeframes between individuals (S1 Fig) [22]. This resulted in 78 samples from 19 individuals (mean = 4.1 samples per individual) for model construction.

### Machine learning models

Read counts were total sum scaled (TSS) by determining the relative abundance of each OTU and normalizing to a standard library size (10,000 for all samples) using phyloseq [48] (v1.44.0). This allowed for comparison of reads across samples and between biomarkers. We also removed OTUs with less than 10 reads across all samples in TSS normalized count tables to reduce noise in the datasets. 16S and ITS TSS read count tables were generated at the phylum, order, and class levels by summing the corresponding OTU table at each respective taxonomic level and then applying the TSS normalization as described above. Taxonomic levels were chosen to represent a subset which covered the full range from phylum to OTU. One of our goals was to compare predictability of bacterial (16S only), fungal (ITS only), or both (16S-ITS) communities; therefore, after TSS normalization, 16S-ITS combined datasets for each taxonomic level (phylum, order, class, OTU) were generated by merging respective 16S and ITS TSS count tables. As a result, 12 datasets were created and used for random forest models.

We chose to apply random forest regression to datasets to predict PMI in ADH. This kept our study similar to those previously conducted on decomposition residues collected from soil surfaces [28, 29], while also assessing predictability of soil microbial succession during human decomposition in our geographical region (Knoxville, TN). Model construction was completed in R using the Ranger [49] (v0.16.0) package. First, samples were assigned to testing or training datasets. This was completed by randomly assigning 6 donors (~1/3) to the test set, while the remaining 13 were grouped into the training set. This approach was conducted following Belk et al. (2018) [28], to ensure that all samples from a single individual were in either the testing or training set, respectively, to prevent overfitting.

Next, random forest regressions were applied to microbial taxa TSS normalized count tables in Ranger. First, random forest model parameters node size (3, 5, 7, 9) and sample size (0.55, 0.632, 0.70, 0.80) were hyper-tuned by comparing models with different combinations of the parameters listed. The optimal model was chosen by assessing the out-of-bag mean square error (OOB MSE) of each model and choosing the set of parameters with the lowest OOB MSE. The optimum model for each biomarker and taxonomic level was assessed by calculating the OOB MSE of the model and the root mean square error (RMSE) and mean absolute error (MAE) for predictions of the testing set in 100 runs of the optimum model. RMSE and MAE were calculated using rmse() and mae() functions from the R package Metrics (v 0.1.4). This process was repeated for models including measured environmental parameters, with values for ambient temperature (°C), pH, electrical conductivity (EC), moisture, *β*-glucosidase (BG) activity, N-acetyl-*β*-D-glucosaminidase (NAG) activity, leucine amino peptidase (LAP) activity, and alkaline phosphatase (PHOS) activity included as model features (Table 1). For all environmental parameters, aside from temperature, log response ratio normalized values [22, 50] were used to account for natural seasonal differences in these parameters. The top 25 most influential model features were extracted from each optimum model to assess taxa/environmental factors influencing model predictions. To evaluate the potential differences in model predication between biomarkers and taxonomic levels, linear regression was applied to the average MAE values (mean of 100 runs per model). Variation in MAE due to treatment variables was assessed with ANOVA, while differences between treatment groups were determined with post-hoc t-tests in R. Code for generating all feature tables and random forest model development can be found at https://github.com/jdebruyn/TOX-microbiology.

**Table 1:** Overview of variables used to construct models. OTU = Operational taxonomic unit.

Type of data	Predictor variables tested
Bacterial and fungal taxa (OTUs) relative abundances	16S OTUs, ITS OTUs, both 16S and ITS OTUs
Phylogenetic level	Phylum, order, class, OTU
Environmental parameters	Ambient temperature, soil electrical coductivity, pH, moisture, enzyme activities (*β*-glucosidase, N-acetyl-*β*-D-glucosaminidase, leucine amino peptidase, and alkaline phosphatase)

## Results

### Soil environmental parameters

We previously reported how the measured soil parameters were altered in response to human decomposition [22]. In summary, soil EC increased with progression of decomposition in soils surrounding all decomposing individuals. Soil pH was variable between individuals, with pH increasing (n = 5 individuals), decreasing (n = 12), or displaying minimal change relative to the controls (n = 2) [22]. Extracellular enzyme activities were also variable between individuals, however general trends included increased NAG and PHOS over time. BG and LAP were variable over time;LAP activity correlated to soil pH [22].

### General model statistics

**Fig 1:**
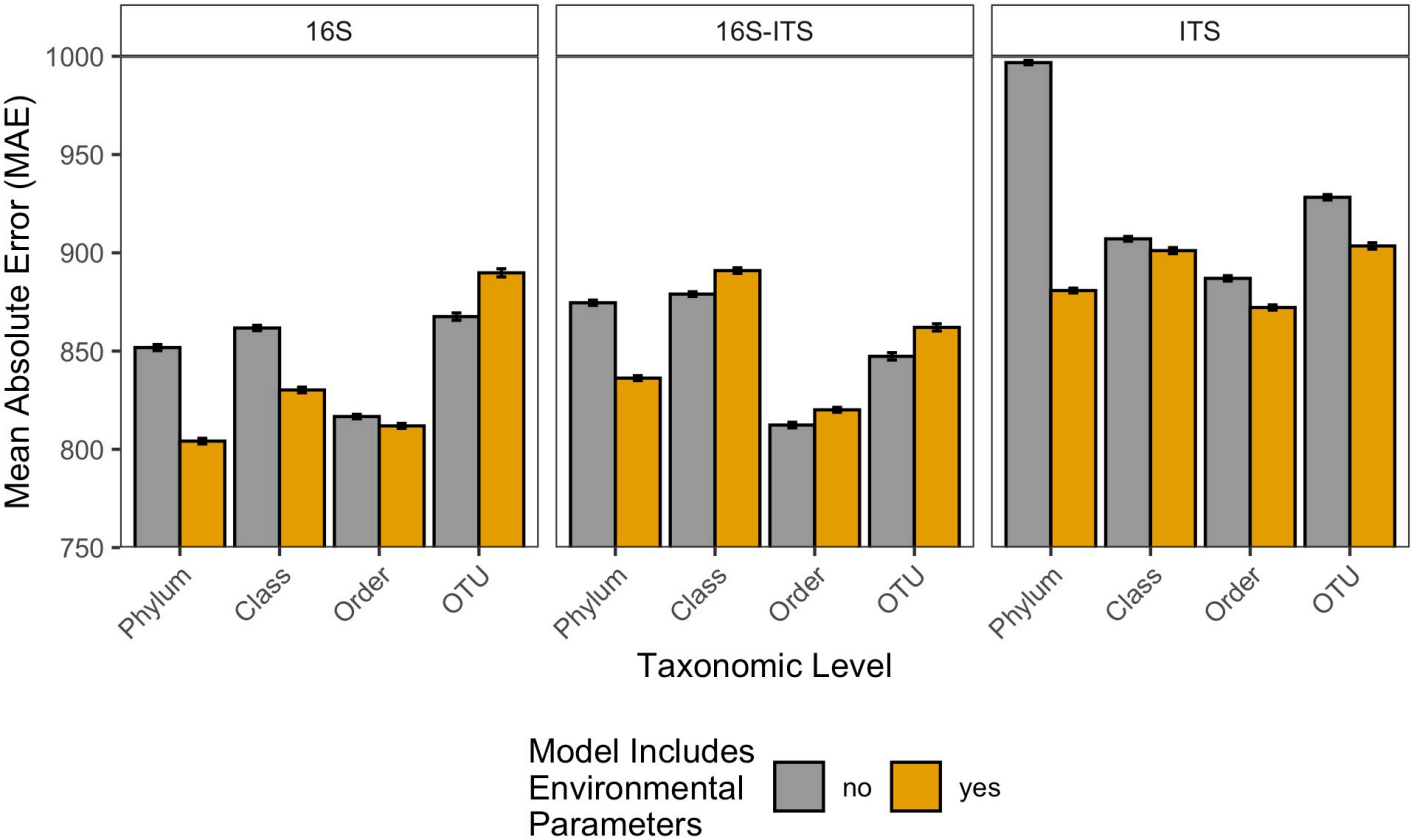
Mean absolute error (MAE) from 100 iterations of each respective model against the testing dataset. Data are reported by biological marker (column), while color compares models with (gold) and without (gray) environmental predictors. Error bars are the standard error of MAE values across all 100 iterations for each respective model.

**Fig 2:**
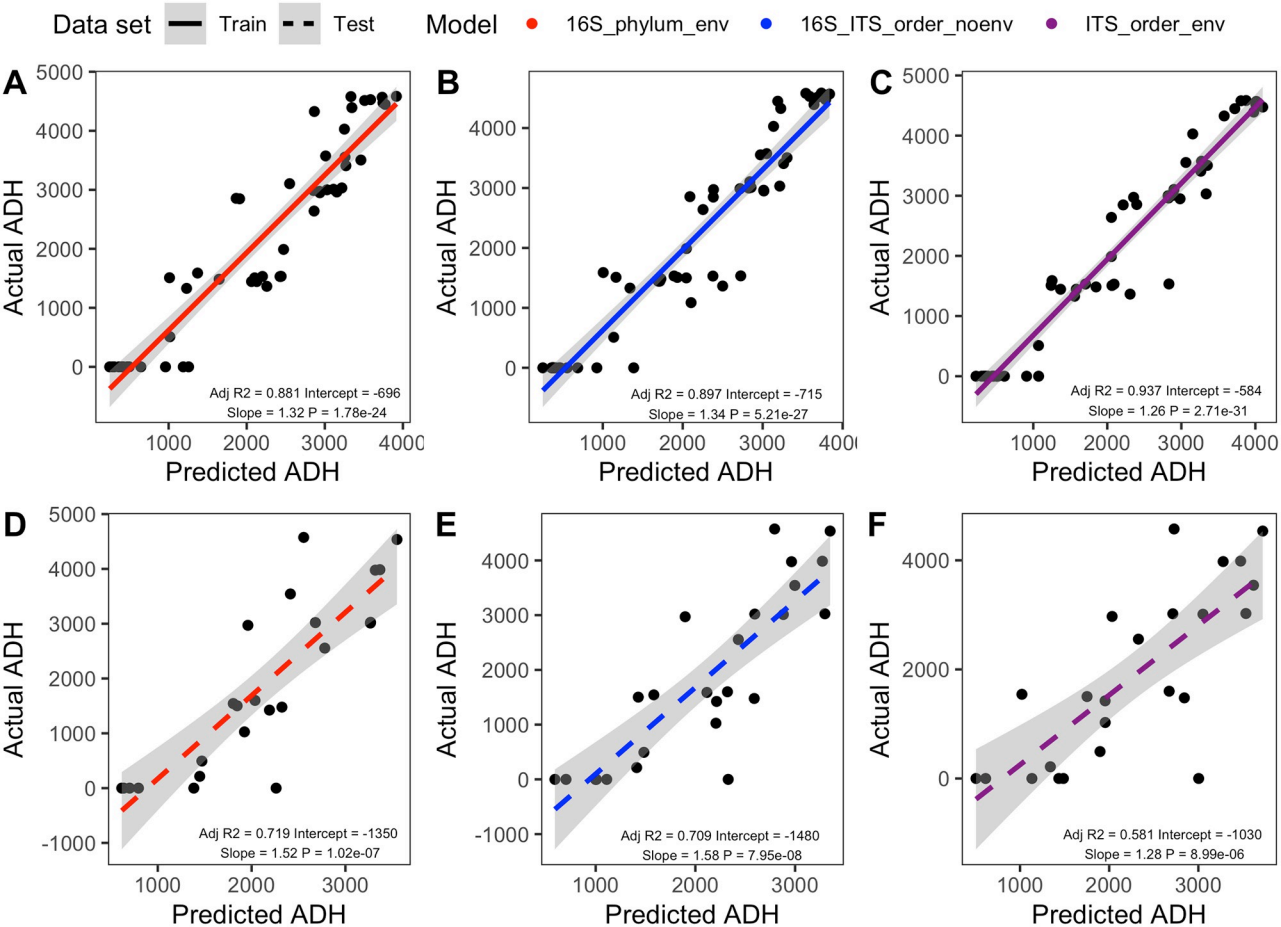
Model predictions for the training set (A-C) and testing set (D-F) for the top performing model for each biological marker as determined by the lowest MAE. For each biological marker, top models included the 16S phylum + environmental data (A, D, line color—red) 16-ITS order (B, E, line color—blue), and ITS order + environmental data (C, F, line color—magenta). Predictability of each model is greater for the training set (A-C) compared to the testing set (D-F). Soild (training set) and dashed (testing set) lines show the best fit linear relationship and shading indicted the 95% confidence interval between actual PMI, in ADH, and predicted ADH within each respective dataset.

In total, 24 models were built in R. Twelve of the models contained environmental features and the other twelve did not. The number of taxa included as features in models without environmental data are reported in Table 2. Bacterial (16S) and fungal (ITS) features ranged from 35 to 5195 and 16 to 2219, respectively, depending on taxonomic level. For all models, MAE ranged from 804.18 to 996.8 ADH (Fig 1). Across all variables considered, the best performing model was the 16S phylum level model with environmental predictors (MAE 804.18) (S2 Table). In contrast, the worst performing model was the ITS phylum level without environmental data (MAE 996.8) (S2 Table). Predictability, assessed by the linear relationship between predicted and observed values, for the training (Fig 2A–2C) and testing (Fig 2D–2F) datasets for the best 16S (phylum + environmental data), ITS only (order + environmental data), and 16S-ITS (order) models are shown in Fig 2. R^2^ for all models ranged from 0.869 to 0.962 when predicting PMI for the training set, however these values were reduced when making predictions for the testing dataset (r^2^ = 0.369–0.741) (S3 Table).

**Table 2:** Number of microbial taxonomic features provided as input for random forest regression investigated in this study. 16S-ITS microbial features are the sum of 16S and ITS features.

	16S Features	16S-ITS Features	ITS Features
OTU	5195	7414	2219
Order	264	411	147
Class	111	177	66
Phylum	35	51	16

### Model comparison: Biological marker

**Fig 3:**
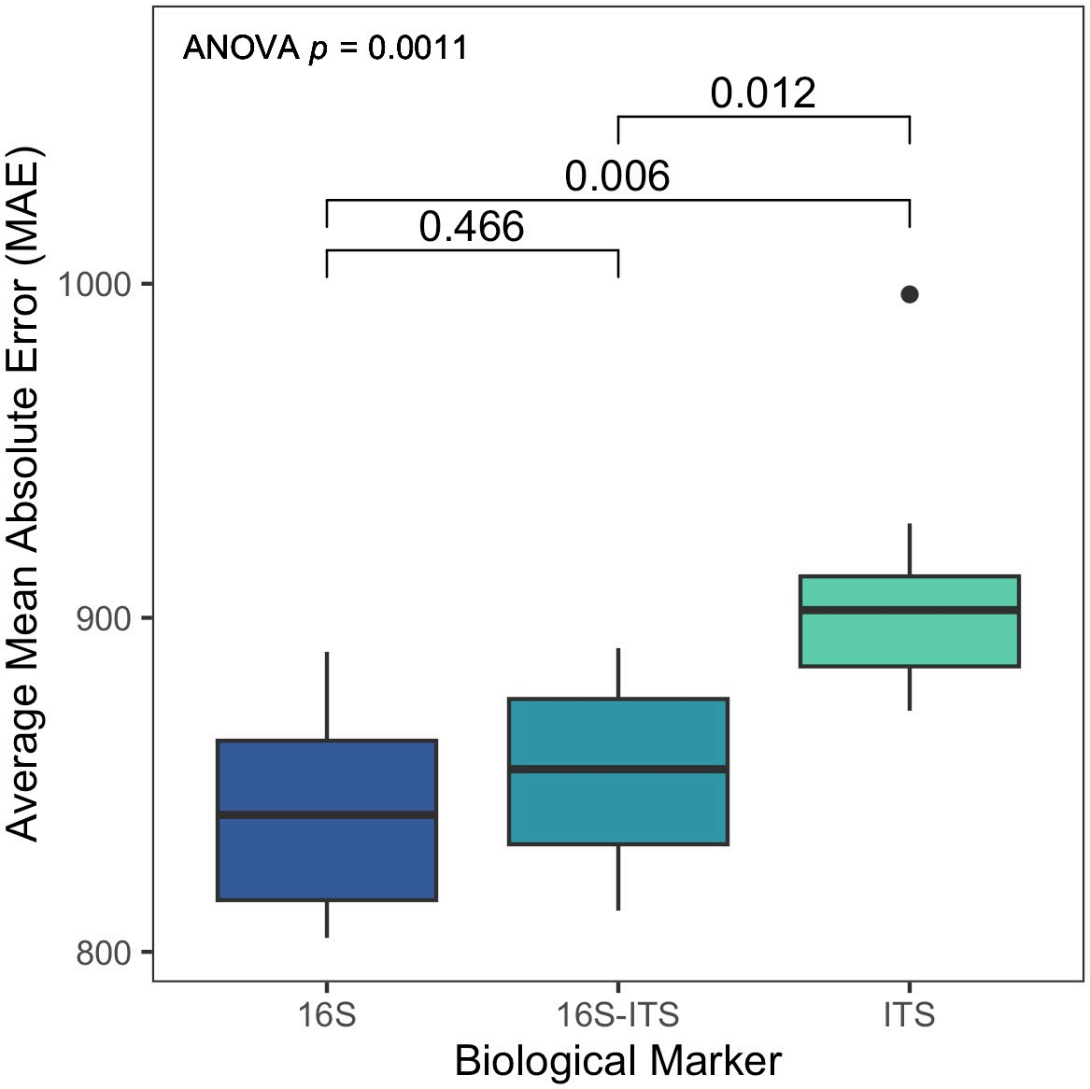
Mean absolute error (MAE) varies as a result of biological marker (16S, 16S-ITS, or ITS) used for model construction. Average MAE is the result of 100 iterations of the 24 respective models against the testing set. Reported p-values are the result of post-hoc t-tests adjusted for multiple comparisons with the Holm method.

Ability of random forest regressions to predict ADH varied depending on the biological marker used to build models (ANOVA F = 9.655, *p* = 0.001) (S4 Table). ITS models were generally less accurate in predicting ADH compared to 16S or 16S-ITS models independent of taxonomic level and environmental data (Fig 3). ITS models ranged in MAE from 872.16 to 996.8 ADH, with a mean MAE of 909.59 ADH. Post-hoc t-tests show that ITS models, in general, had higher MAE than both 16S (t-test *p* = 0.006) and 16S-ITS (*p* = 0.012) models (S5 Table). ITS models represented seven of the 10 worst performing models. In comparison, 16S and 16S-ITS models performed similarly (*p* = 0.466) (S5 Table). 16S models ranged in MAE from 804.18 to 889.81 ADH, with an average MAE of 841.73 ADH, while 16S-ITS models ranged in MAE from 812.35 to 890.94 ADH (average MAE = 852.82 ADH). This can also be observed among the best and worst performing models, where no ITS-only model was in the top 10 best performing models and combined 16S-ITS models were dispersed among the best and worst models. For example, the 16S-ITS order level model without environmental data had the third lowest MAE (MAE 812.35) overall, but also the 16S-ITS class level model with environmental data had the sixth highest MAE (MAE 890.94).

### Model comparison: Taxonomic level

**Fig 4:**
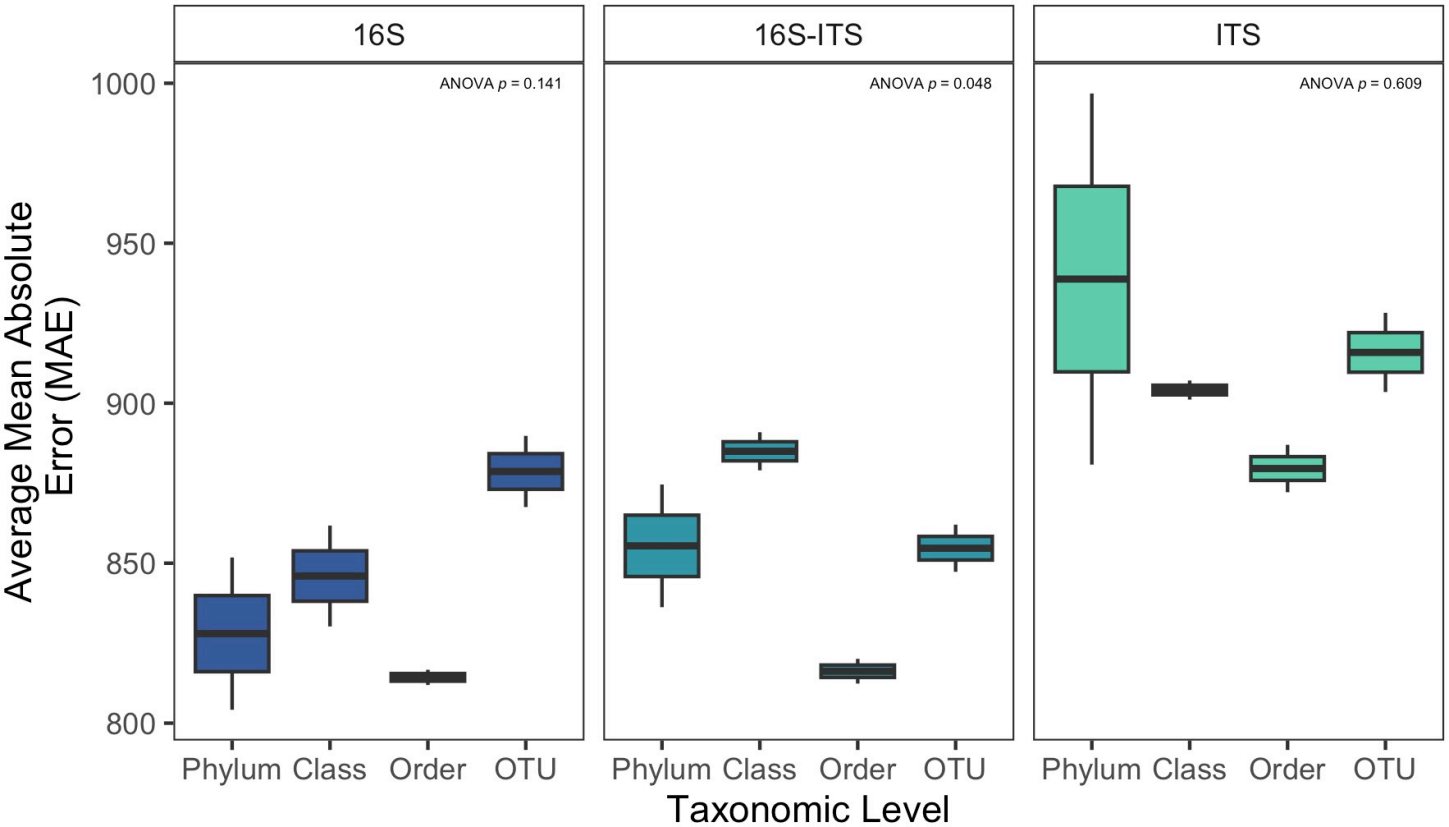
Mean absolute error (MAE) did not vary as a result of taxonomic level (color) used for model construction for any of the biological markers assessed (column). Mean MAE is the result of 100 iterations of the 24 respective models against the testing set. Order level models generally had the lowest MAE, compared to phylum, class, and OTU models. ANOVA p-values are the result of linear models comparing mean MAE to taxonomic level.

Some variation was observed in MAE due to taxonomic level considered for model development, however these differences were not significant (ANOVA F = 1.538; *p* = 0.24) (S6 Table). When considering the potential influence of taxonomic level within biomarkers, no significant difference in MAE by taxonomic level was observed for 16S (*p* = 0.141) or ITS (*p* = 0.609) models, while 16S-ITS models was significant (*p* = 0.048) (S7 Table), likely driven by a difference in MAE between order and class level models for this biological marker (Fig 4). While most results were not significant, some trends were observed. First, order level models had the lowest MAE for all three biological markers assessed. This was also observed in Fig 1, where order level models had the lowest MAE for all models without environmental data. Trends for the other taxonomic levels varied depending on the biological marker in consideration. Phylum and class level models performed similarly within 16S models, with OTU models generating the highest MAE. Within 16S-ITS models, class and OTU level models performed similarly, displaying the first and second highest MAE, respectively. For ITS models, phylum level models had the highest MAE, followed by OTU and class.

### Model comparison: Environmental parameters

Overall, inclusion of environmental parameters in random forest models to predict ADH from soil microbial taxa impacted model accuracy. The direction of effect (*i.e*., increase or decrease in MAE) was dependent on biological marker and taxonomic level considered (Fig 1). For ITS models, inclusion of environmental factors reduced MAE irrespective of the taxonomic level. This reduction was most pronounced for the phylum level model, in which MAE was reduced by 116.007 ADH. In models containing 16S sequencing data (16S and 16S-ITS), effect of environmental features differed by taxonomic level. Specifically, for 16S models, phylum, class, and order level models performed better and OTU level models performed worse when environmental data was included. This was similar for the combined 16S-ITS datasets at the phylum and OTU levels; however, class and order level models performed worse (*i.e*., increased MAE) when environmental factors were included.

### Model features: Top models

**Fig 5:**
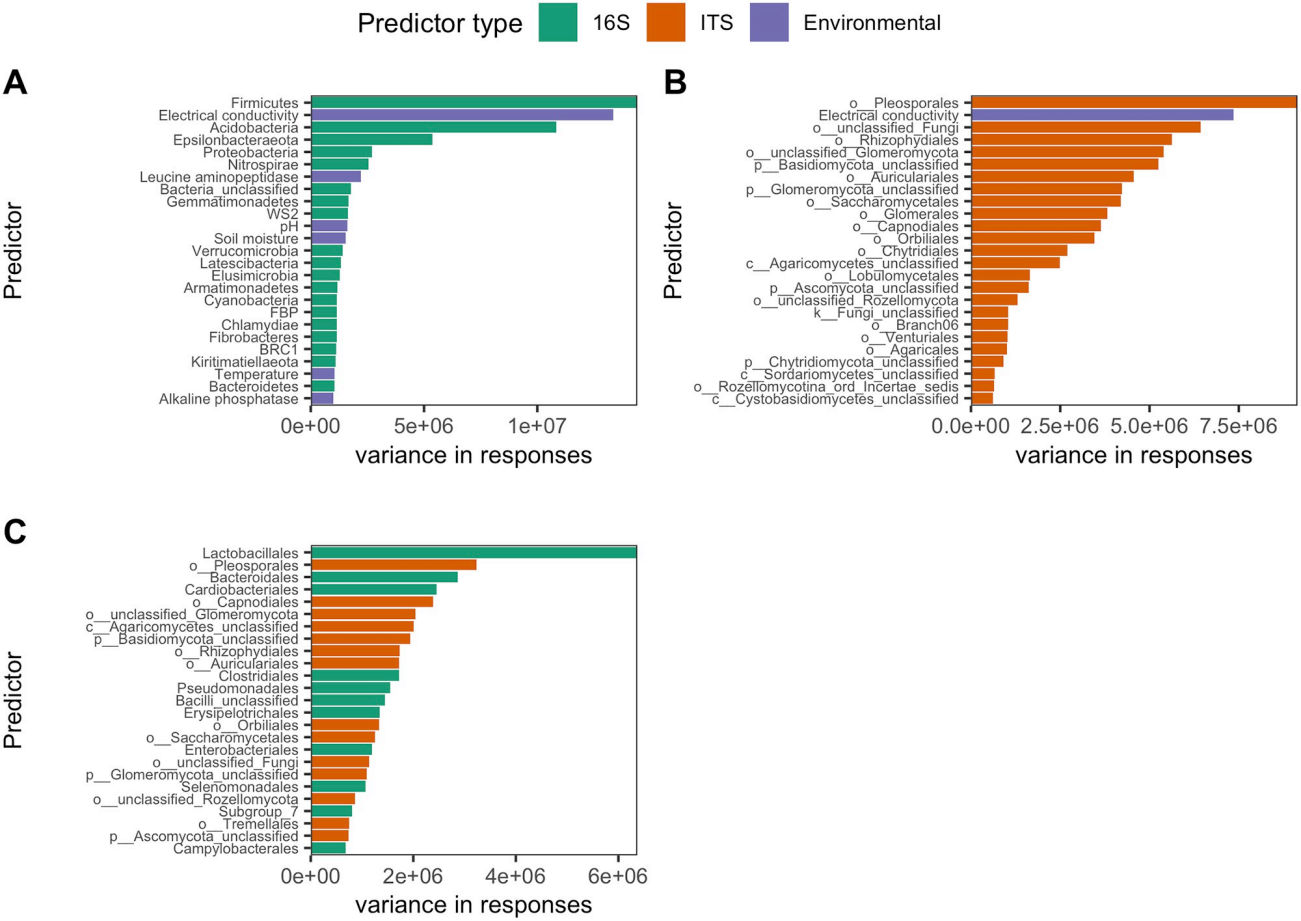
Top 25 model features determined by variance of responses in Ranger. For each biological marker, top models included 16S phylum + environmental data (A), ITS order + environmental data (B), and 16S-ITS order (C). Bar color denotes whether the feature is a 16S taxon (green), ITS taxon (orange), or environmental feature (purple).

In addition to assessing the predictability of different random forest models, we also looked at important model features to observe which taxa and/or environmental parameters impacted model performance. Here we highlight the top 25 features of best performing 16S (phylum + environmental), 16S-ITS (order), and ITS (order + environmental) models (S8 Table), determined by lowest MAE. Both 16S and ITS best models included environmental predictors, while the combined 16S-ITS model did not (Fig 5A–5C). For the 16S phylum model with environmental data, the most important model feature, as assessed by decrease in MSE, was the phylum *Firmicutes*. The remaining important features included soil electrical conductivity (EC), *Acidobacteria*, *Epsilonbacteraeota*, and *Proteobacteria*, respectively. Other features of interest for this model included *Nitrospirae*, leucine aminopeptidase activity, pH, and soil moisture (Fig 5A). For the ITS order model with environmental predictors, the most important model features were *Pleosporales*, soil EC, Unclassified fungi, *Rhizophydiales*, Unclassified *Glomeromycota*, Unclassified *Basidiomycota*, and *Auriculariales* (Fig 5B). In this model, no other environmental parameters were among the top 25 important features. Other top taxonomic features of interest included *Saccharomycetales* and Unclassified *Sordariomycetes*, as their members are present in the human mycobiome and feces, respectively [51, 52]. The best performing 16S-ITS model was the order level model without environmental features. Top features for this model were the bacterial order *Lactobacillales* and the fungal order *Pleosporales*. Bacterial orders *Bacteroidales*, *Cardiobacteriales*, and *Clostridiales* were third, fourth, and eleventh most important features, respectively (Fig 5C). The fungal order *Saccharomycetales* was also observed in the top 25.

**Fig 6:**
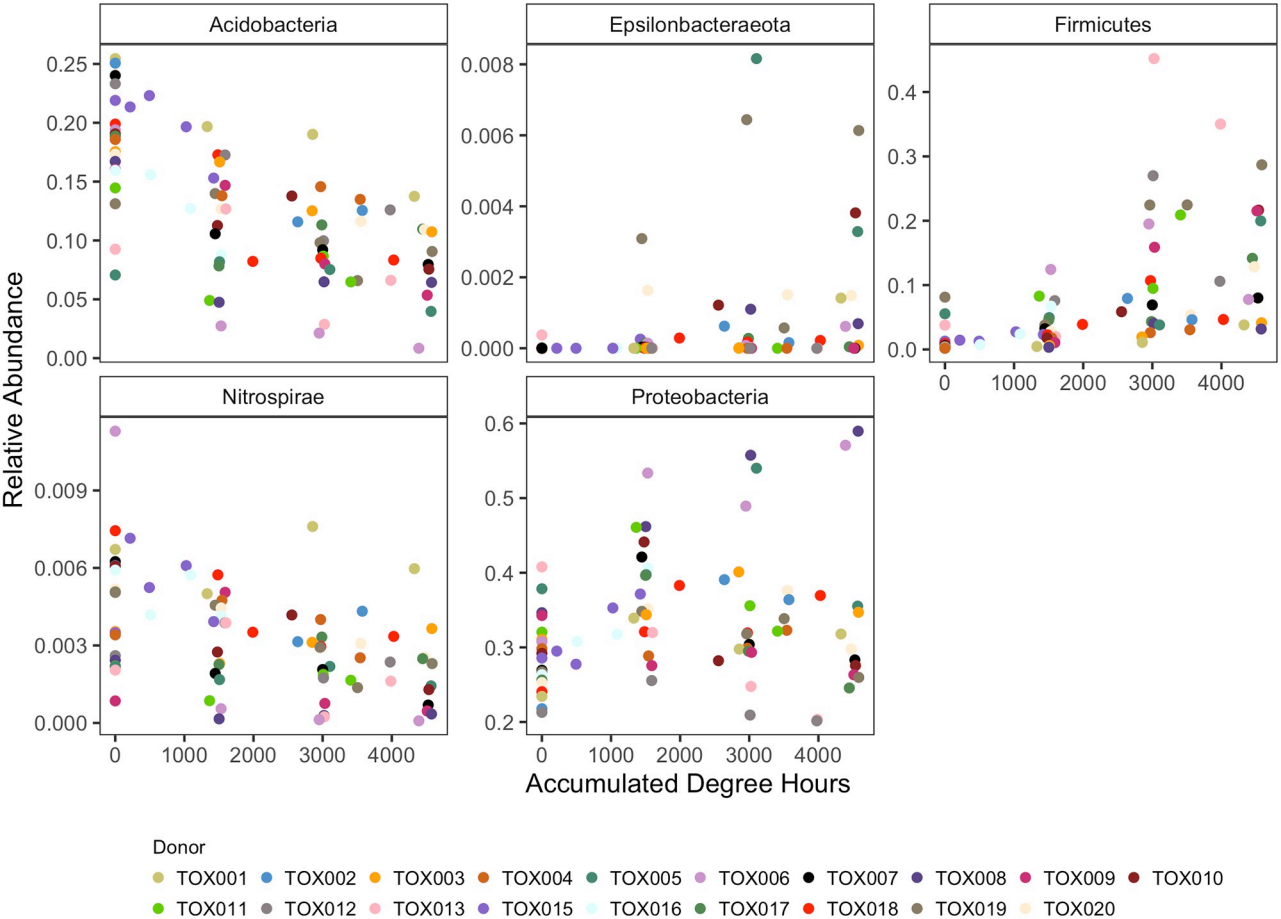
Relative abundance of the 5 most important bacterial phyla in the top 16S random forest model (16S phylum + environmental data). Relative abundance of the phyla Firmicutes, Acidobacteria, Epsilonbacteraeota, Proteobacteria, and Nitrospirae change over time, here accumulated degree hours (ADH), within decomposition-impacted soils. Abundances for each of the 19 individuals (named “TOX###”) are delineated by color.

**Fig 7:**
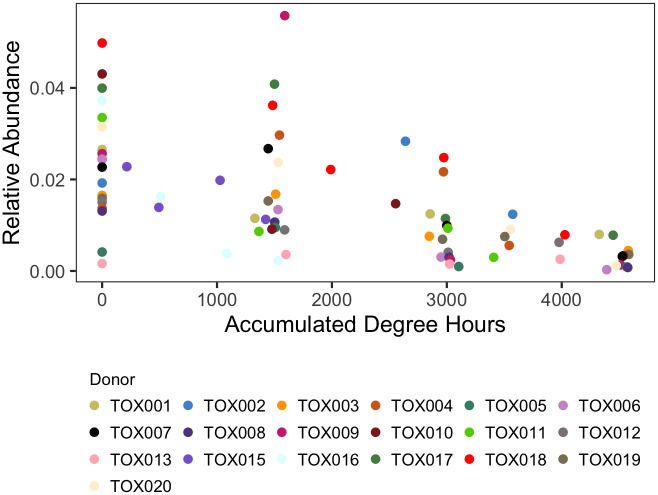
Relative abundance of the fungal order *Pleosporales* over time, here accumulated degree hours (ADH). Abundances for each of the 19 individuals (named “TOX###”) are delineated by color.

Relative abundance of anaerobic bacterial taxa identified in random forest models, including *Firmicutes* (Fig 6), *Bacteroidales* (S2 Fig), *Clostridiales* (S2 Fig) and *Lactobacillales* (S2 Fig), increased as decomposition progressed. In contrast, relative abundance of the aerobic nitrifying organisms of the phylum *Nitrospirae* decreased (Fig 6). *Acidobacteria*, among the top phyla in the 16S model, decreased in relative abundance during decomposition (Fig 6). The phylum *Epsilonbacteraeota*, containg many gut-related taxa, displayed increased relative abundance over time (Fig 6). Relative abundance of the bacterial orders *Cardiobacteriales* and *Pseudomonadales* (S2 Fig) and fungal order *Pleosporales* (Fig 7), identified in the mixed 16S-ITS order model, increased and decreased, respectively.

## Discussion

The goal of this work was to assess the influence of biological marker, taxonomic level, and environmental parameters on model prediction of PMI from soil microbial communities. Model analysis revealed differences between model accuracy due to the biological marker, taxonomic level, and environmental parameters considered for model construction. Overall, models did not predict the test data well. R^2^ dropped from 0.869—0.962 when predicting the training dataset to 0.369—0.741 for the test set. Additionally, models ranged in MAE from 804.18 to 996.8 ADH. In East Tennessee, these error rates would correspond to roughly 2.5 to 3.5 days in July and greater than 28 days in February, based on average seasonal temperatures for the region. Therefore, error rates in the summer would be comparable to those previously reported for microbial communities from organic residues collected from the soil surface (two to six days) [28] but would be substantially higher if considering decomposition during cooler seasons. Further, considering our total decomposition time of 5000 ADH, errors of 804.18 to 996.8 ADH equates to 15.9% to 19.9% of the total decomposition time. The wide error range when including a greater number of subjects across multiple seasons suggests soil microbiome-based models may have low accuracy, particularly when considering individuals across the cline of human variation and through multiple seasons. Specifically, the decomposition systems were influenced by the starting resource, which is dictated by human variation at both the genetic and environmental levels. As a result, intrinsic factors have the capacity to alter both decomposer communities and decomposition rate, and therefore decomposer communities, leading to variation that can impact future models.

One important source of variation in our study was the different rates of decomposition between individuals. While we attempted to correct for differences due to thermal energy input by using accumulated degree hours (ADH), there was still variability in terms of the morphological stage for a given ADH. For example, 5000 ADH represented the end of active decomposition for individual 009, but only about 25% of the active decomposition period for individual 010. Additionally, this time-period did not include decomposition past active decay for any individual in our study, including advanced decomposition or sustained mummification or skeletonization, which could further impact model accuracy. Both individuals (009 and 010) were placed within the facility in the summer, experiencing the same local environmental conditions and potential for insect and scavenger communities, suggesting there are additional factors leading to variation in microbial communities within decomposition-impacted soils. These may include additional environmental parameters not considered in our models, and/or intrinsic differences between the individuals themselves (*e.g*., age, weight, medications, medical conditions etc.) that directly or indirectly impacted microbial communities through interactions with other decomposers (insects and scavengers). Moving forward, we will need to employ a strategy to combine antemortem and environmental data in order to investigate which factors help improve model predictions.

### Influence of diversity and taxa succession on PMI estimations

The trends we observed in model MAE between different biological markers, taxonomic levels, and inclusion of environmental data may be partly explained by differences in diversity between bacterial and fungal (16S vs. ITS) communities and the number of taxa (*i.e*., features), ultimately impacting resolution for predicting PMI. Overall, Chao1 richness and Inverse Simpson diversity were 10 and 15 times lower, respectively, in fungal communities compared to bacterial communities [22]. This translated to differences in the total number of model features for 16S and ITS models: 16S models had roughly 1.7 to 2.3 times more features, depending on the taxonomic level considered. As a result, more features, or taxa, in the dataset with relationships to time (*i.e*., progression of decomposition) may help to distinguish between timepoints to improve model predictability.

In our previous work, we observed that the fungal community composition became more similar as decomposition progressed, with only a few taxa driving fungal successional patterns [22]. This was also observed in Fu et al. (2019) [53], in which only a few taxa (*e.g*., *Ascomycota* sp., *Yarrowia lipolitica*, etc.) displayed relationships with PMI. While we hypothesized that ITS-based models would be more accurate than 16S-based models because of these studies, our results revealed that 16S models generally outperformed ITS-based models and combining 16S and ITS did not improve 16S models alone. This result coincides with those reported by Belk et al. (2018) [28], in which 16S models (mean MAE 4.022 days) had lower error than ITS (mean MAE 4.452 days) or 18S models (mean MAE 4.195 days). The reduced number of taxa observed in fungal communities, in combination with relatively few taxa changing in abundance, may explain why ITS models had higher MAE than 16S models. This may also explain why combining 16S and ITS datasets, which would increase overall diversity, did not outperform either marker alone. With only a few fungal taxa displaying changes over time, their inclusion may not have added additional resolution to the bacterial model.

Diversity differences between bacterial and fungal communities may also drive some of the trends observed between taxonomic levels and with or without environmental factors. In this study, order level models performed best for all biological markers when not considering environmental features. This contrasts with findings by Belk at al. (2018) [28], where lower error was reported for phylum and class level models. This may be linked to a balance between taxonomic resolution and noise for this timeperiod of decomposition. In our study, OTU level models displayed the highest MAE for all biological markers when not considering environmental data, which corresponds with previous decomposition studies reporting increased inter-individual variation at lower taxonomic levels [18, 22, 54]. This may explain why OTU level models displayed the highest MAE for all biological markers when not considering environmental data. Within decomopsition studies, microbial taxonomic succession has mostly been characterized at higher taxonomic levels, at which general patterns are more similar between individuals. However, aggregating microbial abundances at coarse taxonomic levels, such as phyla and class, inherently reduces data dimensionality. It is possible that this decrease in features, in conjunction with trends in taxon abundance over time, reduces the ability of the random forest regression to resolve timepoints at the highest taxonomic levels.

This balance between diversity and features with resolution over time may also explain the effect of environmental features effect on model MAE. We hypothesized that inclusion of environmental parameters would improve all model predictions, by combining soil chemical and microbial successional patterns. While inclusion of environmental predictors improved some models, it decreased performance of others. This effect appears to be linked to biological marker and taxonomic level considered for model creation. Specifically, inclusion of environmental parameters into the lower diversity fungal models added features that helped to improve overall resolution to predict PMI. In contrast, inclusion of environmental data may have added additional noise to high diversity bacterial datasets at lower taxonomic levels, overall leading to decreased model performance.

### Model features

In addition to assessing model performance, we also investigated model features for each top performing 16S, 16S-ITS, and ITS model as determined by lowest MAE. This included the 16S phylum model with environmental data, the 16S-ITS order level model, and the ITS order level model with environmental data. Top model features for 16S phylum level models included taxa observed in previous human and animal decomposition studies, such as the bacterial phyla *Firmicutes*, *Acidobacteria*, and *Proteobacteria* [5, 18]. In our study, *Firmicutes* and *Nitrospirae* were shown to decrease as decomposition progressed, while *Acidobacteria* decreased and *Proteobacteria* remained consistent. These changes seem to be linked to differences in metabolism and environmental changes that occur when decomposition products are released into the surrounding soil. For example, it has been suggested heterotrophic microbial activity responding to the pulse of decomposition products results in depletion of soil oxygen [54, 55]. This would impact the presence of anaerobic gut and soil taxa. While we did not measure soil oxygen in this study, soil respiration was increased in these soils, and so oxygen depletion is to be expected [22]. The increased presence of taxa containing facultative and obligate anaerobic members *Firmicutes* and *Clostridiales* in phylum and order models, respectively, and decrease in *Nitrospirae*, containing nitrifying bacteria that oxidize nitrogen under aerobic conditions, support this hypothesis. Increases in *Firmicutes* and *Clostridiales* follow successional trends observed in internal (*e.g*., organs) microbial communities. Specifically, increased relative abundance of *Clostridium* has been termed the “Clostridium Effect” by Javan et al. (2017) [56] and observed in various organs [6, 56] and the rectum [57] postmortem. Multiple studies, including this current work, have observed increased relative abundance in *Firmicutes* and *Clostridiales* in soils following deposition of decomposition fluid [5, 7, 21, 58], suggesting some of these organisms may be host-derived. Decreased presence of *Acidobacteria* in decomposition-impacted soils is likely linked to their oligotrophic characteristics in response to high nutrient deposition [5, 59]. Succession of these taxa and other taxa past 5000 ADH and the potential implications for PMI models is unclear.

Order level models also revealed some information about soil microbial succession during human decomposition. The 16S-ITS order level model had the lowest MAE among all 16S-ITS models. Within this model, important taxa were a combination of 16S and ITS features present in respective models. Among the top bacterial features, *Lactobacillales*, *Bacteroidales*, and *Clostridiales* were all shown to display general increases as decomposition progressed. This is consistent with previous literature [5]. One interesting find was *Cardiobacteriales* as the fifth most important model feature. *Cardiobacteriales* is a bacterial order of gram-negative rods, whose members are generally capable of fermentation of various sugars [60]. Within this order, only the genus *Ignatzschineria* was identified based on the SILVA non-redundant database (v132) [45]. This genus has been identified in previous outdoor decomposition studies focusing on gut [57], skin [15], and soil [5] microbial communities.*Ignatzschineria* are associated with insect species [61, 62] and first appear in the soil during release of fluid. We previously observed this taxon in bacterial decomposition fluid communities [22], suggesting decomposition fluids as potential vehicle for the transfer of both host- and insect- associated microbes into the surrounding soil. Their association with insects highlights the potential for decomposer insect and scavenger activity to impact microbial succession during decomposition and suggests that PMI estimation models specific to decomposition setting (indoor or outdoor) may be required.

Within the ITS order level models, the fungal order *Pleosporales* was among the most influential taxon for PMI estimation. *Pleosporales*, a member of *Ascomycota* fungi, decreased as decomposition progressed. This was similar to observations by Fu et al. (2019) [53], where *Pleosporales* sp. was shown to be associated with non-decomposition soils by LEfSe (linear discriminant analysis effect size). *Pleosporales* are often associated with plants, found as endophytes, epiphytes, and the rhizosphere [60]. Reduced relative abundance of these fungi in decomposition soils is interesting considering *Pleosporales* have been shown to positively respond to nitrogen amendments [63, 64]. Their response to decomposition products may suggest sensitivity to highly concentrated nitrogen amendments and/or other soil changes, such as osmotic stress in response to high EC, or intolerance to hypoxia typically observed in decomposition soils.

Of the environmental predictors assessed, electrical conductivity (EC) appeared to be most influential. EC was recorded as the top and second most important feature for the 16S phylum and ITS order models with environmental data, respectively. This is likely due to patterns in soil EC being more consistent between individuals over time. Specifically, EC was shown to increase within decomposition soil over time for all 19 individuals. Increases in EC observed in decomposition soils has been shown to positively correlate with increased ammonium concentrations [55, 65], suggesting ammonium would also be a valuable predictor of microbial community dynamics. The other measured environmental parameters (pH, enzyme rates) were not identified as a top predictive features in the models. This is likely because these parameters were more variable both over time as well as between individuals, displaying both increases and decreases in response to human decomposition [22]. While we did not consider all possible environmental parameters in this study, these results suggest that feature selection may help to identify relevant environmental parameters for model construction.

### Limitations and considerations

While there are intriguing investigations suggesting that microbial succession could be used to predict PMI, validation is critical prior to forensic application. Variation between decomposition studies, including vertebrate species observed, and experimental design, along with small sample sizes have limited model development to date. Additionally, most decomposition studies focus on bloat and active decay stages, when decomposers are most active in degrading soft tissues [66, 67]. While informative for initial compositional shifts, this timeframe does not allow us to assess for how long these communities may be impacted or if they return to pre-decomposition conditions [1]. This study starts to address factors that influence PMI estimations from soil microbial succession during human decomposition, however many foundational questions remain. Below we discuss multiple areas to be expanded upon with future investigation.

First, we did not include all possible environmental and soil data as model predictors, nor account for interactions with other decomposer communities. Other factors, such as respiration rates, oxygen concentration, ammonium, nitrate, dissolved organic carbon and nitrogen, sulfur, among others may be relevant features for models predicting PMI within the soil environment as they have been shown to change during decomposition [5, 18, 22, 31–34, 37, 55, 68–74] and have the capacity to structure microbial communities. In addition, it is possible that changes in soil parameters during human decomposition differ based on region due to soil type and climatic differences impacting decomposition progression or presence of microbial taxa [75], as well as the insect and scavenging species present across ecosystems. Lines of inquiry should include, but are not limited to, regional and seasonal (both within and between regions) soil microbial successional patterns in response to carcass decomposition and microbial-insect interactions, including effects of local insect species on microbial community dynamics. For example, *Chrysomya megacephala*, an invasive fly species that has a proclivity for feces, has only recently been documented colonizing human remains in Tennessee, USA [76]. This species carries up to 10 times the pathogenic bacterial load compared to the house fly, *Musca domestica*, potentially introducing microbes that could alter the progression of decomposition and result in different microbial community succession between regions with and without this fly species [77].

Second, we chose to implement the random forest regression algorithm as it is not as sensitive to non-linear data and has high interpretability compared to other forms of supervised and unsupervised machine learning algorithms [78]. This allowed us to assess prediction of PMI and identify taxa and environmental features that correlate with PMI, as well as kept our results similar to previous decomposition studies within the soil environment [28]. However, recent studies have compared multiple machine learning algorithms in other decomposition microhabitats (*i.e*., skin, organs), showing variation both between internal organs [26] and within the same habitat [17]. Both Liu et al. (2020) [26] and Johnson et al. (2016) [17] observed other machine learning algorithms performed better than random forest in higher diversity microhabitats such as the skin and caecum. As the soil environment is among the most diverse microbial habitat on the planet, it is necessary to assess different machine learning approaches when predicting PMI within this microhabitat [79].

Third, total PMI (5000 ADH) considered for model construction will likely impact the performance and applicability of these models [79]. Here we showed that order level models had the lowest MAE in models that do not include environmental features. This contrasts with findings by Belk at al. (2018) [28] and our hypothesis, in which we expected lower error for phylum and class level models, suggesting differences between studies, such as region, sampling strategy, number of individuals, species, study timeframe or intrinsic differences between donor populations may impact model performance. For example, our study went through 5000 ADH, while Belk et al. (2018) [28] presented data up to 25 days. In our study, 5000 ADH corresponded to 13 to 115 days depending on the individual and time of year, suggesting the unit of time chosen for PMI estimates may impact model interpretation. While out of the scope of this paper, a comparison of model performance trained with different units of time would be informative. Additionally, Belk et al. (2018) [28] observed decreased model error when only using data points from the first 25 days of decomposition compared to the first 50 days, suggesting microbial-based models may not be as accurate at higher PMIs. Therefore, future work is needed to determine the PMI range for which microbial-based PMI estimations are most accurate.

Fourth, we chose to use operational taxonomic units (OTUs) and ADH calculated with a baseline of 10°C, as opposed to amplicon sequence variants (ASVs) and/or ADH with a baseline of 0°C or 4°C. The recent application of denoising methods to generate ASVs has become popular in microbial studies using amplicon sequencing. However, we chose to cluster sequences into OTUs to reduce dimensionality in our raw dataset and the probability of splitting single genomes across multiple ASVs [47]. While Glassman and Martiny (2021) [80] observed similar results for alpha and beta diversity from OTUs and ASVs in leaf litter communities, other studies have shown differences in diversity when comparing the two methods [81]. Thus, it is unclear if using OTUs or ASVs will impact machine learning algorithms such as random forest to predict PMI. Future work should investigate differences in PMI estimations from models constructed with OTUs as well as ASVs. Additionally, we chose to use a baseline of 10°C, which is commonly used for entomological methods due to the developmental threshold of regional (east Tennessee) insects [39]. However, other decomposition studies within the soil environment have also used 0°C or 4°C as thresholds for ADH or accumulated degree day (ADD) calculations. These differences may impact PMI estimates; however, no one has addressed effects of different thresholds for ADH/ADD calculation on PMI estimates from microbial successional patterns within the soil. Therefore, a comprehensive comparison between different thermal energy unit (*i.e*., ADH, ADD and baseline) calculations is necessary.

## Conclusion

This study aimed to assess microbial abundance-based prediction of PMI from soil microbial communities. We compared models with different biological markers, taxonomic levels, and presence/absence of environmental variables to expand upon previous estimations of PMI from machine learning algorithms. From this dataset of 19 individuals across multiple seasons, we observed higher error rates and decreased model precision compared to previously published models based on small datasets. Our results show that 16S and 16S-ITS models performed similarly and outperformed ITS models. Further, order level models have the lowest MAE when not considering environmental parameters. We also show that the addition of other factors, such as environmental parameters, have the potential to impact PMI estimations. We observed some level of predictability in soil microbial succession, however high error rates were seen across 19 individuals and across seasons. While our the number of individuals in our study is one of the largest to date, it was demographically limited, and we certainly did not capture all antemortem conditions which could influence decomposition rates. Together this means microbial-based PMI models would need considerable validation and refinement across a diverse population and geographical regions prior to implementing in a forensic context.

## Supporting information

S1 Fig Total decomposition time differs for each donor. Soil samples (black points) were collected at predetermined intervals through the end of active decomposition. Endpoints differed between donors, therefore a cutoff of 5000 ADH (dashed blue line) was chosen to capture the most timepoints across all donors.
(TIF)

S2 Fig Relative abundance of the 5 most important bacterial orders in the top 16S-ITS random forest model (16S-ITS order). Relative abundance of the orders Lactobacillales, Bacteroidales, Cardiobacteriales, Clostridiales, and Pseudomonadales change over time, here accumulated degree hours (ADH), within decomposition-impacted soils. Trends for each of the 19 individuals (named “TOX###”) are delineated by color.
(TIF)

S1 Table Demographics of study individuals. ‘Timepoints for Models’ is the number of samples included in model creation for respective individuals.
(PDF)

S2 Table Summary statistics for all random forest models. Values are means for 100 runs of each model. OOB MSE = out-of-bag mean squared error, RMSE = root mean squared error, MAE = mean absolute error, OTU = Operational taxonomic unit.
(PDF)

S3 Table Cross validation results for all random forest models. Random forest models perform better (r^2^) on training set than the testing set.
(PDF)

S4 Table Analysis of variance (ANOVA) results from linear model testing for the effect of biological marker (*e.g*., 16S, ITS, or 16S-ITS) on random forest model mean absolute error (MAE). (PDF)

S5 Table *Post-hoc* t test results for testing differences between biological marker groups (*e.g*., 16S, ITS, or 16S-ITS). P values were adjusted for multiple comparison (Adjusted *p*) using the Holm method.
(PDF)

S6 Table Analysis of variance (ANOVA) results from linear model testing for the the effect of taxonomic level (*e.g*., phylum, class, order, OTU) on random forest model mean absolute error (MAE). (PDF)

S7 Table Analysis of variance (ANOVA) results from linear models testing for the effect of taxonomic level (*e.g*., phylum, class, order, OTU) within biological marker groups (*e.g*. 16S, ITS, or 16S-ITS) on random forest model mean absolute error (MAE). (PDF)

S8 Table Top 25 most important model features within the top performing model for each biological marker (16S phylum + env, 16S-ITS order, and ITS order + env). Features are 16S OTU (Otu####), ITS OTU (ITS####), or environmetal predictors, depending on the model. Importance reports the the decrease in mean square error (MSE) for each feature. For 16S and ITS features, taxonomy is report to the lowest taxonomic level for each respective model.
(PDF)
