# Supplementary figures and images for: Environmental predictors impact microbial-based postmortem interval (PMI) estimation models within human decomposition soils
Source: PLoS One. 2024 Oct 11;19(10):e0311906. doi: 10.1371/journal.pone.0311906 (PMC11469530; doi:10.1371/journal.pone.0311906)

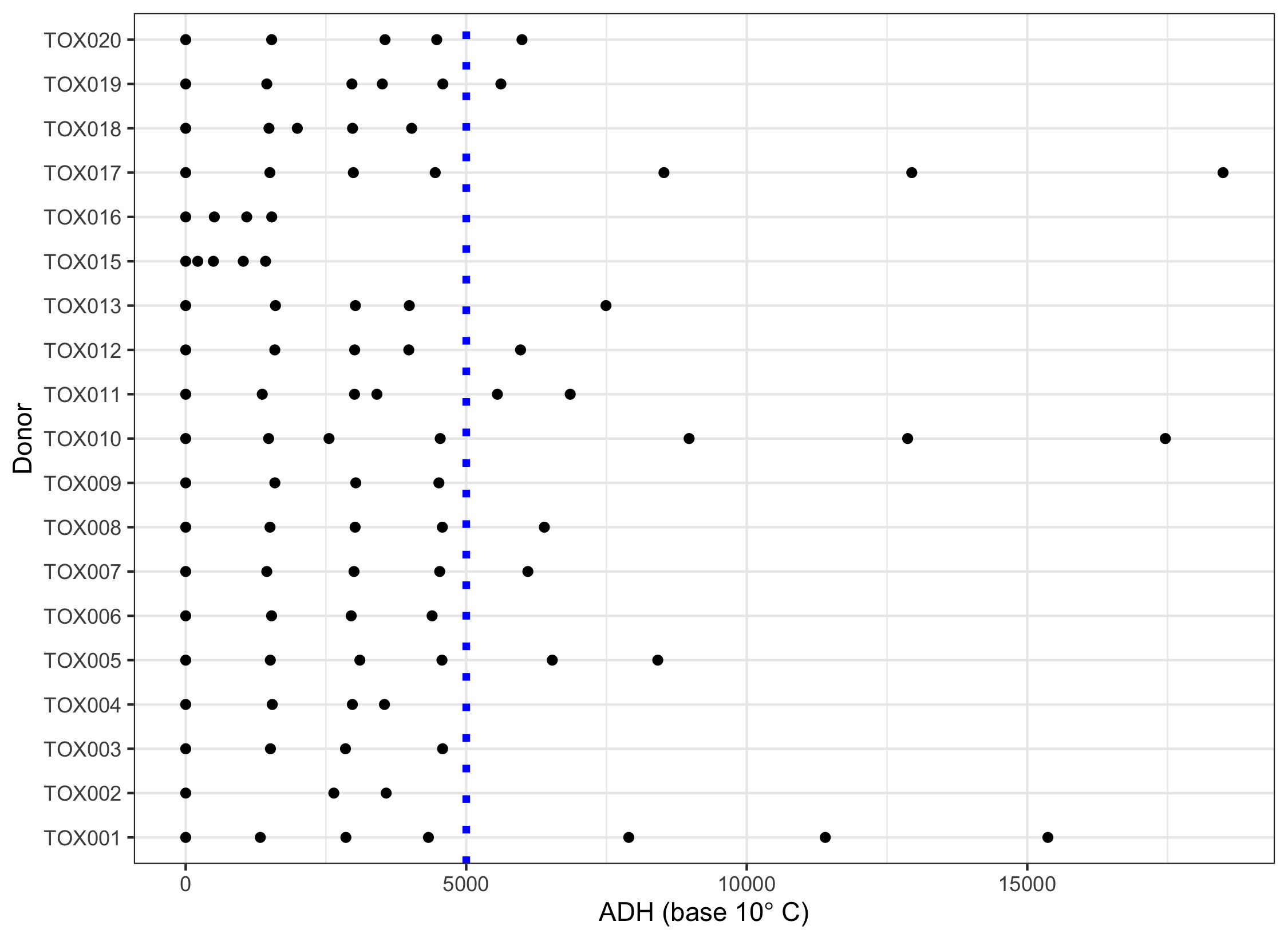

Supplement: S1 Fig — Soil samples (black points) were collected at predetermined intervals through the end of active decomposition. Endpoints differed between donors, therefore a cutoff of 5000 ADH (dashed blue line) was chosen to capture the most timepoints across all donors. (TIF) [file pone.0311906.s001.tif]

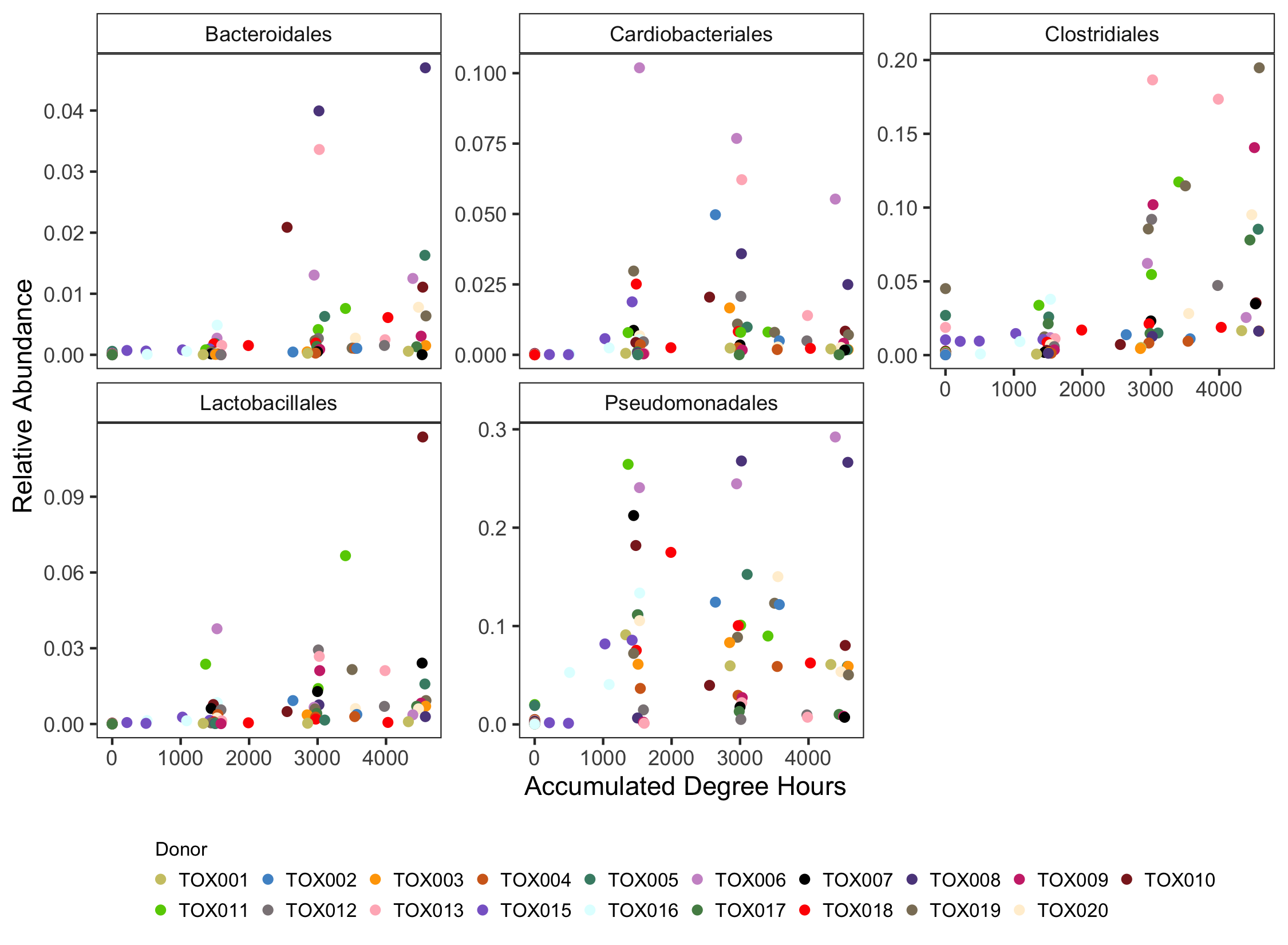

Supplement: S2 Fig — Relative abundance of the orders Lactobacillales, Bacteroidales, Cardiobacteriales, Clostridiales, and Pseudomonadales change over time, here accumulated degree hours (ADH), within decomposition-impacted soils. Trends for each of the 19 individuals (named “TOX###”) are delineated by color. (TIF) [file pone.0311906.s002.tif]
