# Supplementary material for: Environmental predictors impact microbial-based postmortem interval (PMI) estimation models within human decomposition soils
Source: PLoS One. 2024 Oct 11;19(10):e0311906. doi: 10.1371/journal.pone.0311906 (PMC11469530; doi:10.1371/journal.pone.0311906)
Supplement: S1 Table — ‘Timepoints for Models’ is the number of samples included in model creation for respective individuals. (PDF) [file pone.0311906.s003.pdf]

Donor	Age	Sex	Weight	Season	Total ADH	Timpoints in Models
TOX001	Female	63	214	Winter	15500	4
TOX002	Female	78	117	Spring	3750	3
TOX003	Male	71	127	Spring	4500	4
TOX004	Female	84	92	Spring	3500	4
TOX005	Female	71	126	Spring	8500	4
TOX006	Male	64	144	Spring	4500	4
TOX007	Male	89	185	Spring	6000	4
TOX008	Male	40	137	Summer	6500	4
TOX009	Female	72	131	Summer	4500	4
TOX010	Male	65	374	Summer	17500	4
TOX011	Male	81	140	Summer	7000	4
TOX012	Female	77	154	Summer	6000	4
TOX013	Male	54	278	Summer	7500	4
TOX015	Male	78	84	Fall	1500	5
TOX016	Male	62	280	Fall	1500	4
TOX017	Female	62	311	Winter	18500	4
TOX018	Female	85	75	Winter	4000	5
TOX019	Female	67	100	Winter	5500	5
TOX020	Male	91	185	Spring	6000	4
