# Supplementary material for: Environmental predictors impact microbial-based postmortem interval (PMI) estimation models within human decomposition soils
Source: PLoS One. 2024 Oct 11;19(10):e0311906. doi: 10.1371/journal.pone.0311906 (PMC11469530; doi:10.1371/journal.pone.0311906)
Supplement: S2 Table — Values are means for 100 runs of each model. OOB MSE = out-of-bag mean squared error, RMSE = root mean squared error, MAE = mean absolute error, OTU = Operational taxonomic unit. (PDF) [file pone.0311906.s004.pdf]

Model name	OOB MSE	RMSE	MAE	r^2	Marker	Taxonomic Level	Environmental parameters
16S phylum env	1444615.5	959.4219	804.1781	0.4655315	16S	Phylum	yes
16S order env	1244806.8	930.0531	811.9157	0.5394553	16S	Order	yes
16S ITS order noenv	1189663.7	967.0769	812.3529	0.5472546	16S-ITS	Order	no
16S order noenv	1255877.9	926.5153	816.6988	0.5220557	16S	Order	no
16S ITS order env	1111697.3	987.1160	820.1087	0.5887022	16S-ITS	Order	yes
16S class env	1431031.2	1001.9648	830.2113	0.4705573	16S	Class	yes
16S ITS phylum env	1177324.2	1047.7942	836.2375	0.5644220	16S-ITS	Phylum	yes
16S ITS otu noenv	1384491.8	1009.7389	847.2944	0.4731096	16S-ITS	OTU	no
16S phlyum noenv	1534529.4	1058.5407	851.7822	0.4160104	16S	Phylum	no
16S class noenv	1527864.0	1055.6984	861.7494	0.4185470	16S	Class	no
16S ITS otu env	1320722.5	1037.3521	862.0506	0.5113686	16S-ITS	OTU	yes
16S otu noenv	1452233.1	1026.6750	867.5294	0.4473296	16S	OTU	no
ITS order env	1103379.9	1086.9547	872.1639	0.5917794	ITS	Order	yes
16S ITS phylum noenv	1258265.1	1093.2208	874.5923	0.5211472	16S-ITS	Phylum	no
16S ITS class noenv	1209584.0	1073.3961	878.9998	0.5396736	16S-ITS	Class	no
ITS phylum env	1150272.6	1117.6971	880.7924	0.5744304	ITS	Phylum	yes
ITS order noenv	1205103.7	1106.3941	887.0036	0.5413786	ITS	Order	no
16S otu env	1427895.5	1053.3246	889.8058	0.4717174	16S	OTU	yes
16S ITS class env	1133573.6	1091.3316	890.9399	0.5806086	16S-ITS	Class	yes
ITS class env	995536.1	1123.5863	901.1152	0.6316787	ITS	Class	yes
ITS otu env	1255170.8	1099.3132	903.4867	0.5356209	ITS	OTU	yes
ITS class noenv	1100420.9	1137.3096	907.1108	0.5812173	ITS	Class	no
ITS otu noenv	1355602.1	1130.8974	928.2569	0.4841040	ITS	OTU	no
ITS phylum noenv	1427807.1	1253.3553	996.7999	0.4566253	ITS	Phylum	no
