# Supplementary material for: Environmental predictors impact microbial-based postmortem interval (PMI) estimation models within human decomposition soils
Source: PLoS One. 2024 Oct 11;19(10):e0311906. doi: 10.1371/journal.pone.0311906 (PMC11469530; doi:10.1371/journal.pone.0311906)
Supplement: S3 Table — Random forest models perform better (r2) on training set than the testing set. (PDF) [file pone.0311906.s005.pdf]

Model	Dataset	Adjusted r^2	Intercept	Slope	p-value
ITS phylum noenv	train	0.894	-604	1.28	0.00e+00
ITS phylum noenv	test	0.369	-113	0.91	7.69e-04
ITS phylum env	train	0.880	-582	1.27	0.00e+00
ITS phylum env	test	0.547	-548	1.08	2.22e-05
ITS otu noenv	train	0.954	-838	1.35	0.00e+00
ITS otu noenv	test	0.585	-1340	1.38	5.10e-06
ITS otu env	train	0.962	-812	1.34	0.00e+00
ITS otu env	test	0.649	-1520	1.46	1.20e-06
ITS order noenv	train	0.941	-577	1.25	0.00e+00
ITS order noenv	test	0.506	-726	1.21	4.05e-05
ITS order env	train	0.937	-584	1.26	0.00e+00
ITS order env	test	0.581	-1030	1.28	9.00e-06
ITS class noenv	train	0.931	-622	1.27	0.00e+00
ITS class noenv	test	0.521	-877	1.24	2.79e-05
ITS class env	train	0.941	-553	1.24	0.00e+00
ITS class env	test	0.567	-1040	1.26	1.31e-05
16S prune otu noenv	train	0.922	-845	1.40	0.00e+00
16S prune otu noenv	test	0.708	-2020	1.92	1.00e-07
16S prune otu env	train	0.952	-784	1.36	0.00e+00
16S prune otu env	test	0.675	-1990	1.81	5.00e-07
16S phylum env	train	0.881	-696	1.32	0.00e+00
16S phylum env	test	0.719	-1350	1.52	1.00e-07
16S phlyum noenv	train	0.913	-588	1.28	0.00e+00
16S phlyum noenv	test	0.573	-799	1.32	7.20e-06
16S otu noenv	train	0.935	-775	1.36	0.00e+00
16S otu noenv	test	0.707	-2110	1.90	1.00e-07
16S otu env	train	0.949	-755	1.35	0.00e+00
16S otu env	test	0.697	-2020	1.82	2.00e-07
16S order noenv	train	0.943	-568	1.27	0.00e+00
16S order noenv	test	0.740	-1290	1.49	0.00e+00
16S order env	train	0.921	-616	1.29	0.00e+00
16S order env	test	0.741	-1340	1.47	0.00e+00
16S class noenv	train	0.878	-795	1.39	0.00e+00
16S class noenv	test	0.596	-1210	1.52	3.70e-06
16S class env	train	0.902	-762	1.35	0.00e+00
16S class env	test	0.686	-1470	1.58	4.00e-07
16S ITS phylum noenv	train	0.930	-518	1.24	0.00e+00
16S ITS phylum noenv	test	0.490	-437	1.08	5.90e-05
16S ITS phylum env	train	0.869	-571	1.26	0.00e+00
16S ITS phylum env	test	0.576	-636	1.14	1.04e-05
16S ITS otu noenv	train	0.945	-800	1.36	0.00e+00
16S ITS otu noenv	test	0.705	-2120	1.84	1.00e-07
16S ITS otu env	train	0.953	-743	1.33	0.00e+00
16S ITS otu env	test	0.705	-1720	1.68	2.00e-07
16S ITS order noenv	train	0.897	-715	1.34	0.00e+00
16S ITS order noenv	test	0.709	-1480	1.58	1.00e-07
16S ITS order env	train	0.935	-629	1.28	0.00e+00
16S ITS order env	test	0.707	-1350	1.46	2.00e-07
16S ITS class noenv	train	0.914	-686	1.32	0.00e+00
16S ITS class noenv	test	0.579	-965	1.34	6.10e-06
16S ITS class env	train	0.894	-756	1.35	0.00e+00
16S ITS class env	test	0.614	-1230	1.39	3.60e-06
