# Supplementary material for: Environmental predictors impact microbial-based postmortem interval (PMI) estimation models within human decomposition soils
Source: PLoS One. 2024 Oct 11;19(10):e0311906. doi: 10.1371/journal.pone.0311906 (PMC11469530; doi:10.1371/journal.pone.0311906)
Supplement: S5 Table — P values were adjusted for multiple comparison (Adjusted p) using the Holm method. (PDF) [file pone.0311906.s007.pdf]

Group 1	Group 2	Statistic	df	p	Adjusted p
16S	16S-ITS	-0.750	13.937	0.466	0.466
16S	ITS	-3.856	13.199	0.002	0.006
16S-ITS	ITS	-3.305	12.782	0.006	0.012
