# Supplementary material for: Environmental predictors impact microbial-based postmortem interval (PMI) estimation models within human decomposition soils
Source: PLoS One. 2024 Oct 11;19(10):e0311906. doi: 10.1371/journal.pone.0311906 (PMC11469530; doi:10.1371/journal.pone.0311906)
Supplement: S6 Table — (PDF) [file pone.0311906.s008.pdf]

	Df	Sum Sq	Mean Sq	F value	Pr(>F)
taxonomicLevel	3	8101.989	2700.663	1.494	0.247
Residuals	20	36156.076	1807.804	NA	NA
