# Supplementary material for: Environmental predictors impact microbial-based postmortem interval (PMI) estimation models within human decomposition soils
Source: PLoS One. 2024 Oct 11;19(10):e0311906. doi: 10.1371/journal.pone.0311906 (PMC11469530; doi:10.1371/journal.pone.0311906)
Supplement: S7 Table — (PDF) [file pone.0311906.s009.pdf]

Biological Marker	Df	Sum.Sq	Mean.Sq	F value	Pr(>F)
16S	3	4647.036	1549.012	3.278	0.141
16S-ITS	3	4765.094	1588.365	6.718	0.048
ITS	3	3645.664	1215.221	0.679	0.609
