# Supplementary material for: Environmental predictors impact microbial-based postmortem interval (PMI) estimation models within human decomposition soils
Source: PLoS One. 2024 Oct 11;19(10):e0311906. doi: 10.1371/journal.pone.0311906 (PMC11469530; doi:10.1371/journal.pone.0311906)
Supplement: S8 Table — Features are 16S OTU (Otu####), ITS OTU (ITS####), or environmetal predictors, depending on the model. Importance reports the the decrease in mean square error (MSE) for each feature. For 16S and ITS features, taxonomy is report to the lowest taxonomic level for each respective model. (PDF) [file pone.0311906.s010.pdf]

Feature	Importance	Type	Kingdom	Phylum	Class	Order
**16S phylum env**						
Otu00024	14417047.0	16S	Bacteria	Firmicutes		
Electrical conductivity	13357604.8	Environmental				
Otu00007	10828905.5	16S	Bacteria	Acidobacteria		
ITS00083	9146151.7	ITS	k___Fungi	p___Ascomycota	c___Dothideomycetes	o___Pleosporales
Electrical conductivity	7355183.3	Environmental				
ITS00236	6437920.0	ITS	k___Fungi	p___unclassified_Fungi	c___unclassified_Fungi	o___unclassified_Fungi
Otu00024	6371154.9	16S	Bacteria	Firmicutes	Bacilli	Lactobacillales
ITS00050	5625491.0	ITS	k___Fungi	p___Chytridiomycota	c___Rhizophydiomycetes	o___Rhizophydiales
ITS00211	5398908.1	ITS	k___Fungi	p___Glomeromycota	c___unclassified_Glomeromycota	o___unclassified_Glomeromycota
Otu00345	5367482.3	16S	Bacteria	Epsilonbacteraeota		
ITS00063	5253131.2	ITS	k___Fungi	p___Basidiomycota	p___Basidiomycota_unclassified	p___Basidiomycota_unclassified
ITS00190	4554279.9	ITS	k___Fungi	p___Basidiomycota	c___Agaricomycetes	o___Auriculariales
ITS00377	4226477.6	ITS	k___Fungi	p___Glomeromycota	p___Glomeromycota_unclassified	p___Glomeromycota_unclassified
ITS00002	4204692.8	ITS	k___Fungi	p___Ascomycota	c___Saccharomycetes	o___Saccharomycetales
ITS00289	3826988.5	ITS	k___Fungi	p___Glomeromycota	c___Glomeromycetes	o___Glomerales
ITS00098	3632691.1	ITS	k___Fungi	p___Ascomycota	c___Dothideomycetes	o___Capnodiales
ITS00250	3459217.0	ITS	k___Fungi	p___Ascomycota	c___Orbiliomycetes	o___Orbiliales
ITS00083	3233614.4	ITS	k___Fungi	p___Ascomycota	c___Dothideomycetes	o___Pleosporales
Otu00151	2866877.4	16S	Bacteria	Bacteroidetes	Bacteroidia	Bacteroidales
Otu00002	2714318.1	16S	Bacteria	Proteobacteria		
ITS00556	2705240.1	ITS	k___Fungi	p___Chytridiomycota	c___Chytridiomycetes	o___Chytridiales
Otu00089	2556199.4	16S	Bacteria	Nitrospirae		
ITS00411	2483909.5	ITS	k___Fungi	p___Basidiomycota	c___Agaricomycetes	c___Agaricomycetes_unclassified
Otu00001	2455280.6	16S	Bacteria	Proteobacteria	Gammaproteobacteria	Cardiobacteriales
ITS00098	2387414.1	ITS	k___Fungi	p___Ascomycota	c___Dothideomycetes	o___Capnodiales
**16S-ITS order noenv**						
Leucine aminopeptidase	2201260.5	Environmental				
ITS00211	2036567.5	ITS	k___Fungi	p___Glomeromycota	c___unclassified_Glomeromycota	o___unclassified_Glomeromycota
ITS00411	2004794.1	ITS	k___Fungi	p___Basidiomycota	c___Agaricomycetes	c___Agaricomycetes_unclassified
ITS00063	1943164.4	ITS	k___Fungi	p___Basidiomycota	p___Basidiomycota_unclassified	p___Basidiomycota_unclassified
Otu00222	1769699.5	16S	Bacteria	Bacteria_unclassified		
ITS00050	1734139.6	ITS	k___Fungi	p___Chytridiomycota	c___Rhizophydiomycetes	o___Rhizophydiales
ITS00190	1726824.0	ITS	k___Fungi	p___Basidiomycota	c___Agaricomycetes	o___Auriculariales
Otu00003	1722296.1	16S	Bacteria	Firmicutes	Clostridia	Clostridiales
Otu00274	1678610.1	16S	Bacteria	Gemmatimonadetes		
ITS00455	1656266.1	ITS	k___Fungi	p___Chytridiomycota	c___Lobulomycetes	o___Lobulomycetales
Otu02148	1638095.8	16S	Bacteria	WS2		
pH	1628692.3	Environmental				
ITS00298	1611808.0	ITS	k___Fungi	p___Ascomycota	p___Ascomycota_unclassified	p___Ascomycota_unclassified
Otu00002	1554846.7	16S	Bacteria	Proteobacteria	Gammaproteobacteria	Pseudomonadales
Soil moisture	1527363.6	Environmental				
Otu00008	1452337.8	16S	Bacteria	Firmicutes	Bacilli	Bacilli_unclassified
Otu00006	1421801.6	16S	Bacteria	Verrucomicrobia		
Otu00383	1340426.9	16S	Bacteria	Firmicutes	Erysipelotrichia	Erysipelotrichales
Otu00313	1338310.0	16S	Bacteria	Latescibacteria		
ITS00250	1329344.5	ITS	k___Fungi	p___Ascomycota	c___Orbiliomycetes	o___Orbiliales
ITS00053	1298390.2	ITS	k___Fungi	p___Rozellomycota	c___unclassified_Rozellomycota	o___unclassified_Rozellomycota
Otu01125	1285801.7	16S	Bacteria	Elusimicrobia		
ITS00002	1254193.8	ITS	k___Fungi	p___Ascomycota	c___Saccharomycetes	o___Saccharomycetales
Otu00022	1190179.5	16S	Bacteria	Proteobacteria	Gammaproteobacteria	Enterobacteriales
Otu01505	1165988.4	16S	Bacteria	Armatimonadetes		
**ITS order env**						
Otu00947	1156528.3	16S	Bacteria	Cyanobacteria		
Otu02192	1154412.8	16S	Bacteria	FBP		
Otu01783	1151594.1	16S	Bacteria	Chlamydiae		
Otu02338	1149628.2	16S	Bacteria	Fibrobacteres		
ITS00236	1135120.9	ITS	k___Fungi	p___unclassified_Fungi	c___unclassified_Fungi	o___unclassified_Fungi
Otu01278	1134895.4	16S	Bacteria	BRC1		
Otu06070	1098996.1	16S	Bacteria	Kiritimatiellaeota		
ITS00377	1091289.0	ITS	k___Fungi	p___Glomeromycota	p___Glomeromycota_unclassified	p___Glomeromycota_unclassified
Otu00039	1067006.2	16S	Bacteria	Firmicutes	Negativicutes	Selenomonadales
Temperature	1054968.4	Environmental				
Otu00025	1049223.3	16S	Bacteria	Bacteroidetes		
ITS00012	1046203.7	ITS	k___Fungi	k___Fungi_unclassified	k___Fungi_unclassified	k___Fungi_unclassified
ITS00157	1035446.4	ITS	k___Fungi	p___Ascomycota	c___Sordariomycetes	o___Branch06
ITS00400	1019423.2	ITS	k___Fungi	p___Ascomycota	c___Dothideomycetes	o___Venturiales
ITS00051	1007171.0	ITS	k___Fungi	p___Basidiomycota	c___Agaricomycetes	o___Agaricales
Alkaline phosphatase	983422.9	Environmental				
ITS00072	911780.8	ITS	k___Fungi	p___Chytridiomycota	p___Chytridiomycota_unclassified	p___Chytridiomycota_unclassified
ITS00053	861529.9	ITS	k___Fungi	p___Rozellomycota	c___unclassified_Rozellomycota	o___unclassified_Rozellomycota
Otu00165	811247.4	16S	Bacteria	Acidobacteria	Holophagae	Subgroup_7
ITS00132	751410.4	ITS	k___Fungi	p___Basidiomycota	c___Tremellomycetes	o___Tremellales
ITS00298	736688.3	ITS	k___Fungi	p___Ascomycota	p___Ascomycota_unclassified	p___Ascomycota_unclassified
Otu00345	685326.0	16S	Bacteria	Epsilonbacteraeota	Campylobacteria	Campylobacterales
ITS00499	664170.6	ITS	k___Fungi	p___Ascomycota	c___Sordariomycetes	c___Sordariomycetes_unclassified
ITS00683	647369.1	ITS	k___Fungi	p___Rozellomycota	c___Rozellomycotina_cls_Incertae_sedis	o___Rozellomycotina_ord_Incertae_sedis
ITS00968	621350.4	ITS	k___Fungi	p___Basidiomycota	c___Cystobasidiomycetes	c___Cystobasidiomycetes_unclassified
